# Significantly Improved Colossal Dielectric Properties and Maxwell—Wagner Relaxation of TiO_2_—Rich Na_1/2_Y_1/2_Cu_3_Ti_4+*x*_O_12_ Ceramics

**DOI:** 10.3390/molecules26196043

**Published:** 2021-10-05

**Authors:** Pariwat Saengvong, Narong Chanlek, Bundit Putasaeng, Atip Pengpad, Viyada Harnchana, Sriprajak Krongsuk, Pornjuk Srepusharawoot, Prasit Thongbai

**Affiliations:** 1 Giant Dielectric and Computational Design Research Group (GD–CDR), Department of Physics, Faculty of Science, Khon Kaen University, Khon Kaen 40002, Thailand; mute1987@gmail.com (P.S.); atippe@kku.ac.th (A.P.); viyada@kku.ac.th (V.H.); srikro@kku.ac.th (S.K.); spornj@kku.ac.th (P.S.); 2 Synchrotron Light Research Institute (Public Organization), 111 University Avenue, Muang District, Nakhon Ratchasima 30000, Thailand; narong@slri.or.th; 3 National Metal and Materials Technology Center, National Science and Technology Development Agency, Thailand Science Park, Pathum Thani 12120, Thailand; bunditp@mtec.or.th; 4 Institute of Nanomaterials Research and Innovation for Energy (IN–RIE), Khon Kaen University, Khon Kaen 40002, Thailand

**Keywords:** colossal/giant dielectric properties, X–ray photoelectron spectroscopy, Maxwell–Wagner relaxation, impedance spectroscopy, NYCTO

## Abstract

In this work, the colossal dielectric properties and Maxwell—Wagner relaxation of TiO_2_–rich Na_1/2_Y_1/2_Cu_3_Ti_4+*x*_O_12_ (*x* = 0–0.2) ceramics prepared by a solid-state reaction method are investigated. A single phase of Na_1/2_Y_1/2_Cu_3_Ti_4_O_12_ is achieved without the detection of any impurity phase. The highly dense microstructure is obtained, and the mean grain size is significantly reduced by a factor of 10 by increasing Ti molar ratio, resulting in an increased grain boundary density and hence grain boundary resistance (*R*_gb_). The colossal permittivities of *ε*′ ~ 0.7–1.4 × 10^4^ with slightly dependent on frequency in the frequency range of 10^2^–10^6^ Hz are obtained in the TiO_2_–rich Na_1/2_Y_1/2_Cu_3_Ti_4+*x*_O_12_ ceramics, while the dielectric loss tangent is reduced to tanδ ~ 0.016–0.020 at 1 kHz due to the increased *R*_gb_. The semiconducting grain resistance (*R*_g_) of the Na_1/2_Y_1/2_Cu_3_Ti_4+*x*_O_12_ ceramics increases with increasing *x*, corresponding to the decrease in Cu^+^/Cu^2+^ ratio. The nonlinear electrical properties of the TiO_2_–rich Na_1/2_Y_1/2_Cu_3_Ti_4+*x*_O_12_ ceramics can also be improved. The colossal dielectric and nonlinear electrical properties of the TiO_2_–rich Na_1/2_Y_1/2_Cu_3_Ti_4+*x*_O_12_ ceramics are explained by the Maxwell–Wagner relaxation model based on the formation of the Schottky barrier at the grain boundary.

## 1. Introduction

Improvement of electronic device efficiency through the development of materials with enhanced electrical properties is significant. Colossal dielectric oxides (CDOs) with very high dielectric constant (*ε*′) are widely used to manufacture critical components in electronic devices, especially for multilayer ceramic capacitors (MLCCs) [1,2,3]. The *ε*′ of a CDO influences the geometry and performance of the MLCCs. The size of an MLCC can be miniaturized by using the insulating oxide between the metallic electrodes with a dielectric oxide with a higher *ε*′ value than the conventional oxide. Many CDOs have intensively been investigated, especially for CaCu_3_Ti_4_O_12_ (CCTO) and related compounds, expecting to replace traditional CDOs such as BaTiO_3_—based ceramics.

The dielectric and electrical properties of the perovskite CCTO ceramics have been extensively studied over the past two decades [4,5,6,7,8,9,10,11,12]. This is because CCTO ceramics showed high *ε*′ ~ 10^3^–10^5^ over wide ranges of temperature and frequency. Moreover, the *ε*′ of CCTO ceramics are relatively stable in the temperature range of 100–400 K compared to conventional BaTiO_3_–based ceramics used. Unfortunately, CCTO still presents too high dielectric loss (tanδ >> 0.05), which is not required for application in MLCCs [13,14,15]. Therefore, researchers have studied reducing the tanδ of CCTO ceramics by tuning the ceramic microstructure of CCTO and related ceramics according to their heterogeneous electrical structure. The special microstructure, which consists of semiconducting grains and highly resistive boundaries (GBs), can be produced using one–step processing method [5]. This heterogeneous microstructure is called to be an internal barrier layer capacitor (IBLC) structure. Accordingly, the resistivity and correlated tanδ can be improved by engineering the grains and GBs [16,17]. In addition, the presence of insulating GBs affects a nonlinear relationship between current density (*J*) and electrical field strength (*E*), which is a behavior required for developing varistor devices [4,9,18].

To improve the colossal dielectric and nonlinear *J*–*E* properties, many effective methods have been proposed and studied, such as doping with suitable ions [19,20,21,22], tuning ceramic microstructure [9] and fabricated CCTO–matrix composites [10,18,23,24]. These methods have the same approach, which is to increase the total resistance of the insulating GBs (*R*_gb_) for reducing tanδ. One of the most effective methods is to fabricate the CCTO–matrix composites using an appropriate ceramic filler such as CaTiO_3_ (CTO), Al_2_O_3_ or TiO_2_ [6,7,10,18,25]. For the CTO/CCTO/and TiO_2_/CCTO composites, although the tanδ can be significantly reduced to <0.05, their *ε*′ values are usually significantly decreased in the order of 10^3^. For these two composite systems, the nonlinear electrical properties can also be significantly improved. The TiO_2_/CCTO composites can be easily prepared by designing TiO_2_–rich phase in the CCTO ceramics using the formula CaCu_3_Ti_4+x_O_12+2x_. Notably, the mean grain size was reduced, resulting in a significant increase in *R*_gb_. This is the primary cause of the observed improvement of the colossal dielectric and nonlinear electrical properties of CCTO ceramics.

In addition to CCTO ceramics, the colossal dielectric properties of ACu_3_Ti_4_O_12_ oxides (A = Na_1/2_Bi_1/2_ [26], Na_1/2_Y_1/2_ [27,28,29,30], Na_1/2_La_1/2_ [31], Bi_2/3_ [32,33], Y_2/3_ [32,34], Cd [16,17,35], La_2/3_ [32], Sm_2/3_ [36], Na_1/3_Ca_1/3_Bi_1/3_ [37], Na_1/3_Cd_1/3_Y_1/3_ [38], Na_1/3_Sr_1/3_Y_1/3_ [39] and Na_1/2_Sm_1/2_ [40]) are very attractive, especially for the Na_1/2_Y_1/2_Cu_3_Ti_4_O_12_ (NYCTO) ceramics [27,28,29,30]. The NYCTO ceramics exhibited a high *ε*′ ~ 10^4^ with low tanδ < 0.05 at 1 kHz compared to that of the CCTO ceramics [28,29,30]. Recently, the preparation, colossal dielectric permittivity and nonlinear electrical properties of NYCTO ceramics has been widely reported [27,28,29,30,39,41,42,43].

In this work, the TiO_2_—rich NYCTO ceramics were prepared by a conventional mixed—oxide method and investigated the dielectric properties. The crystal structure, phase composition and microstructural evolution of the sintered ceramics, as well as their oxidation states, were characterized. The primary cause of the enhanced colossal dielectric response was systematically elucidated. This study contributes an exciting concept for improving the colossal dielectric properties of the NYCTO ceramics by reducing their tanδ. We believe that this research work provides an effective route to improve the CDOs for future applications in MLCCs.

## 2. Experimental Details

TiO_2_—rich Na_1/2_Y_1/2_Cu_3_Ti_4+*x*_O_12_ (NYCTO+xTiO_2_) ceramics (where *x* = 0.0, 0.1 and 0.2) were prepared using solid-state reaction method (SSR). The starting materials were Na_2_CO_3_ (99.9%), Y_2_O_3_ (99.99%), CuO (99.9%) and TiO_2_ (99.9%), which were purchased from Sigma–Aldrich (St. Louis, MO, USA). Details for the preparation of NYCTO—based oxides were provided elsewhere [28,30,39]. The mixed powders for all compositions were calcined at 1000 °C for 10 h. Mixed powders (without calcination) were pressed into 9.5-mm-diameter pellets by uniaxial compression at ~100 MPa. Finally, the pellets were sintered at 1070 °C for 10 h in air. The sintered NYCTO + xTiO_2_ ceramics with *x* = 0.0, 0.1 and 0.2 were referred to as the NYCTO, NYCTO + 0.1TiO_2_ and NYCTO + 0.2TiO_2_, respectively.

The crystal structures of the sintered sample were characterized using X–ray diffraction (XRD, PANalytical, EMPYREAN, Shanghai, China), scanning electron microscopy (MiniSEM, SEC, SNE–4500 M), field emission scanning electron microscopy (FIB–FESEM,) with energy dispersive X–ray (EDX) spectroscopy and X–ray photoemission spectroscope (XPS, PHI5000 Versarobe II, ULVAC–PHI, Chigasaki, Japan). Comprehensive details were provided in our previous works [12,21,44,45,46].

For the nonlinear electrical and dielectric measurements, the surfaces of samples were polished. Next, the parallel and smooth surfaces were coated with silver paints and fired in the air at 600 °C for 30 min. The impedance and dielectric parameters of all sintered ceramics were measured with an impedance analyzer (KEYSIGHT E4990A, Santa Rosa, CA, USA). The dielectric properties were measured in the temperature range of −160 to 210 °C and the frequency range from 10^2^–10^7^ Hz. The nonlinear relationship between current density (*J*) and electrical field strength (*E*) was analyzed by using a high–voltage measurement unit (Keithley 247 model, Cleveland, OH, USA).

## 3. Results and Discussion

**Figure 1:**
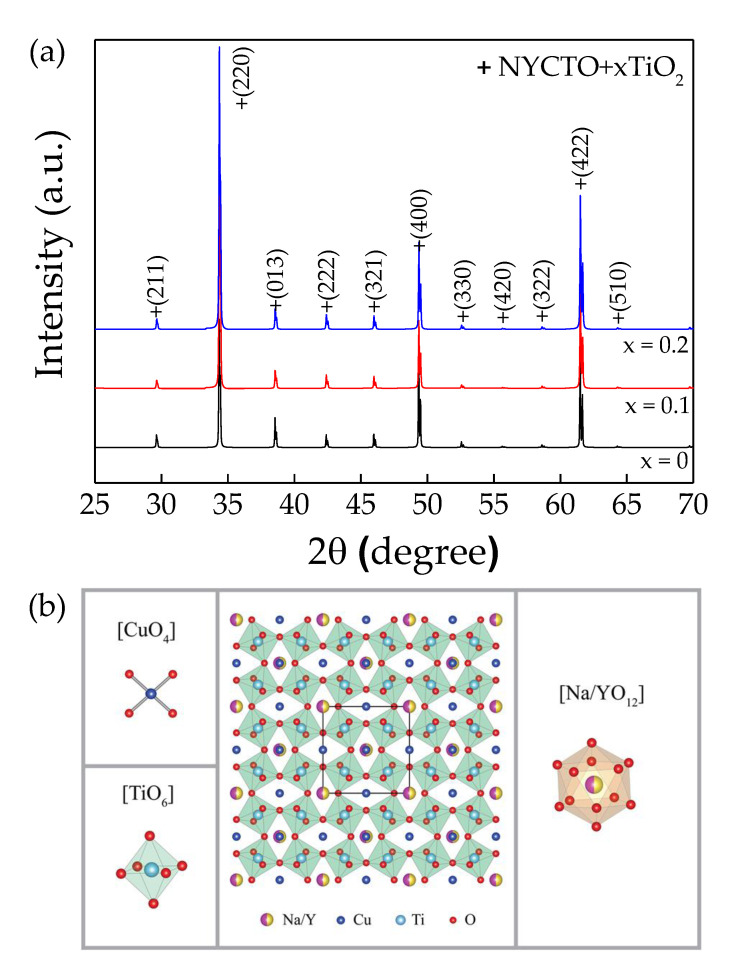
(**a**) XRD patterns of NYCTO + xTiO_2_ ceramics with *x* = 0.0, 0.1 and 0.2. (**b**) NYCTO structure.

The XRD patterns of the sintered NYCTO + xTiO_2_ (*x* = 0, 0.1 and 0.2) ceramics are illustrated in Figure 1a, showing the single phase of NYCTO in all ceramics with a perovskite-structure (JCPDS 75–2188). The crystal structure of NYCTO is demonstrated in Figure 1b. The XRD peak corresponding to TiO_2_ phase cannot be detected in the NYCTO + 0.1TiO_2_ and NYCTO + 0.2TiO_2_ ceramics, which may be due to a small amount of an excess TiO_2_ molar ratio that was lower than the resolution limit of the XRD technique. Accordingly, the lattice parameters (*a*) can be calculated and found to be 7.383, 7.383 and 7.384 Å for the NYCTO, NYCTO + 0.1TiO_2_ and NYCTO + 0.2TiO_2_ ceramics, respectively. The *a* values are comparable to those reported in the literature [27,28,29,30]. The excess TiO_2_ composition in NYCTO ceramics does not affect the lattice parameter. This result indicates that the TiO_2_—rich phase is segregated from the primary phase of NYCTO, which may exist as the TiO_2_—rich boundary. The XRD result shows that the NYCTO + xTiO_2_ has successfully been fabricated using the SSR method.

**Figure 2:**
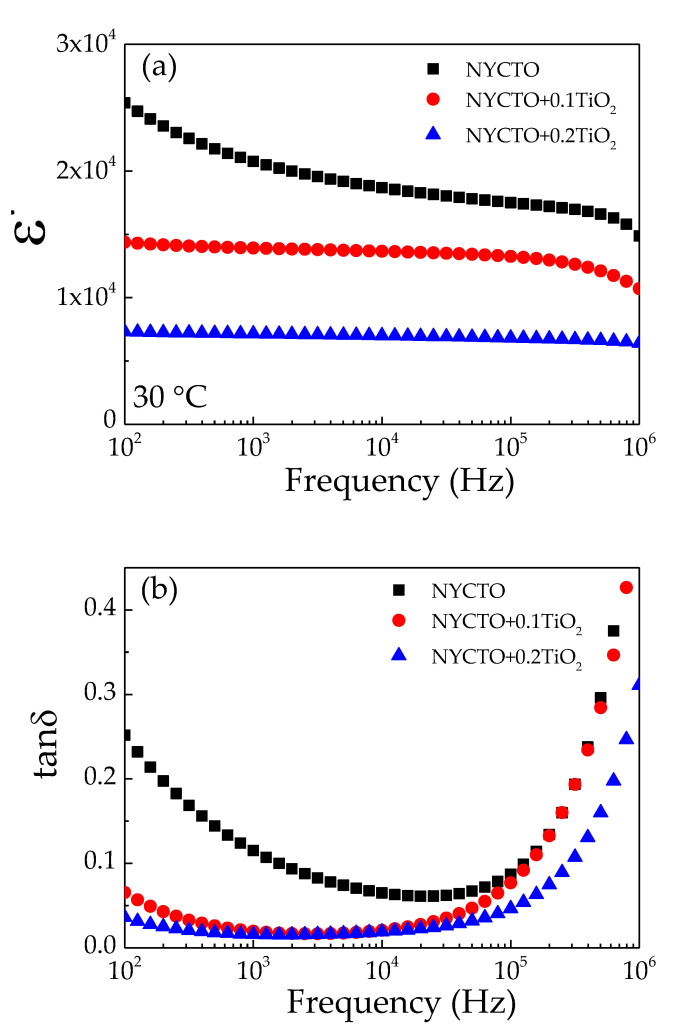
Dielectric properties at room temperature as a function of frequency for NYCTO + xTiO_2_ ceramics with different doping content: (**a**) dielectric permittivity (*ε*′) and (**b**) loss tangent (tanδ).

Even though the excessive TiO_2_ phase was not detected in the XRD patterns for all ceramics, the variation in compositions of the CuO and/or TiO_2_ ratios in an ACu_3_Ti_4_O_12_ compound usually affects the dielectric and electrical properties [6,10,24,45,47,48,49]. Thus, we first investigated the dielectric properties of the NYCTO + xTiO_2_ ceramics at around room temperature (30 °C). The relationship between the *ε*′ and frequency of the NYCTO + xTiO_2_ ceramics is shown in Figure 2a. The *ε*′ value of the NYCTO ceramic is huge (2.07 × 10^4^ at 10^3^ Hz) with a quite low tanδ ~ 0.115, which is similar to that reported in the previous works [27,28,30]. However, the *ε*′ of the NYCTO ceramic is largely dependent on the frequency in a low–frequency range, which is usually owing to the dominant effect of non–Ohmic sample–electrode interface [8,15,44,50]. At 10^6^ Hz, the *ε*′ begins to decline due to the primary dielectric relaxation mechanism [12,19]. Interestingly, the *ε*′ values of the NYCTO + 0.1TiO_2_ and NYCTO + 0.2TiO_2_ ceramics are more stable with frequency than that of the NYCTO ceramic. The TiO_2_—rich phase can improve the frequency dependence of the *ε*′ of the NYCTO + xTiO_2_ ceramics. The *ε*′ values of the NYCTO + 0.1TiO_2_ and NYCTO + 0.2TiO_2_ ceramics at 30 °C and 10^3^ Hz are 1.39 × 10^4^ and 7.15 × 10^3^, respectively. Even though the dielectric response in the NYCTO + 0.1TiO_2_ ceramic was decreased due to the excessive TiO_2_, its *ε*′ value was still larger than 10^4^ over the measured frequency range. The decrease in the *ε*′ value of the NYCTO + xTiO_2_ ceramics is similar to that observed in the CaCu_3_Ti_4+*x*_O_12_ [6].

Figure 2b illustrates the tanδ at 30 °C for the NYCTO + xTiO_2_ ceramics over the frequency range of 10^2^–10^6^ Hz. In the frequency range of 10^2^–10^5^ Hz, the tanδ values of the NYCTO + 0.1TiO_2_ and NYCTO + 0.2TiO_2_ ceramics are much lower than the NYCTO ceramic. Furthermore, in this frequency range, the tanδ values of the NYCTO + 0.1TiO_2_ and NYCTO + 0.2TiO_2_ ceramics are lower than 0.1. The rapid increase in tanδ of all the ceramics is attributed to the primary dielectric relaxation, i.e., Maxwell—Wagner polarization relaxation [12,19]. The tanδ values of the NYCTO, NYCTO + 0.1TiO_2_ and NYCTO + 0.2TiO_2_ ceramics 10^3^ Hz are 0.115, 0.020 and 0.016, respectively. Notably, the tanδ value of the NYCTO ceramics can be significantly reduced by increasing the excessive TiO_2_ molar ratio. This result indicates the influential role of TiO_2_-rich on the significantly improved dielectric properties of the NYCTO ceramics.

**Figure 3:**
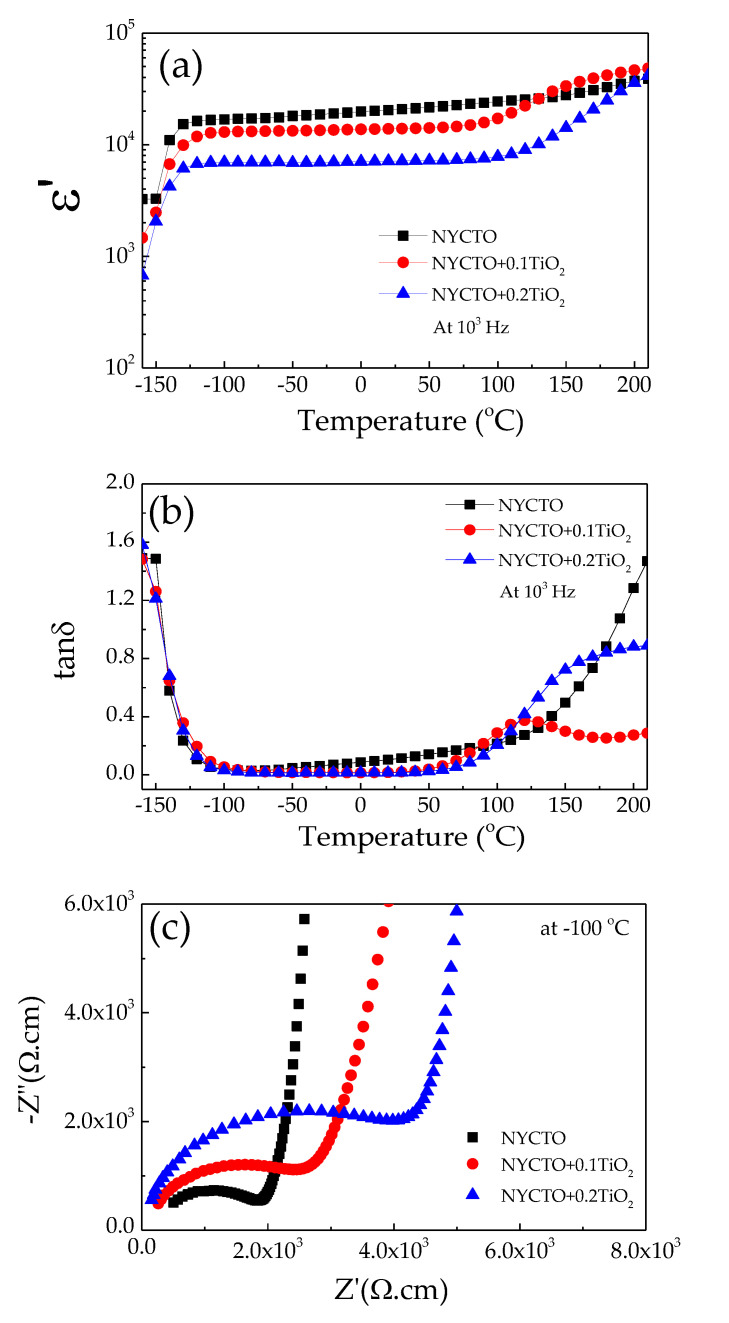
(**a**,**b**) Dielectric permittivity (*ε*′) and loss tangent (tanδ) of NYCTO + xTiO_2_ ceramics as a function of temperature (−150–200 °C). (**c**) Impedance complex plane plots (Z*) at −100 °C, showing the electrical response on semiconducting grains.

The temperature dependence of the dielectric properties of the NYCTO + xTiO_2_ ceramics is illustrated in Figure 3a,b. In the temperature range from −125 to 110 °C, the *ε*′ values of the NYCTO + 0.1TiO_2_ and NYCTO + 0.2TiO_2_ ceramics are more stable with temperature than that of the NYCTO ceramic. At the temperature below −120 °C, the *ε*′ rapidly decreased, corresponding to the rapid increase in tanδ. This is the Maxwell—Wagner polarization relaxation in the NYCTO + xTiO_2_ ceramics. Considering in the high-temperature range, the *ε*′ and tanδ significantly increase, which is associated with the DC conduction of free charge carriers associated with oxygen vacancies [11,33,51,52].

It is widely accepted that the colossal dielectric properties of the ACu_3_Ti_4_O_12_ oxide groups is the result from the electrical heterogeneous in the microstructure [5,11,15,30,33,48]. The electrically heterogeneous microstructure can be confirmed using impedance spectroscopy [5,53]. Furthermore, the heterogeneous electrical microstructure can also be used to explain the nonlinear electrical properties of the ACu_3_Ti_4_O_12_ ceramics [21,23,24]. Generally, the semicircular arc due to the electrical response of the semiconducting grains of the ACu_3_Ti_4_O_12_ compounds can be observed in the impedance complex plane (Z*) plots at low temperatures [5,54]. Thus, to confirm the formation of semiconducting grains, Z* plots of all the NYCTO + xTiO_2_ ceramics are demonstrated at −100 °C, as shown in Figure 3c. The semicircular arcs of all the ceramics can be observed, while only parts of relatively large semicircular arcs can be observed. These two parts can be assigned as the electrical responses of the semiconducting grain and insulating GB, respectively [5,54]. The grain resistance (*R*_g_), which can be calculated from the diameter of the semicircular arc of the NYCTO + xTiO_2_ ceramics, was increased by increasing the TiO_2_ molar ratio. In general, we expect that the TiO_2_—rich should not affect the electrical properties of the semiconducting grains but should only affect the insulating boundaries due to the segregation of the TiO_2_—rich phase. In this current study, the excessive TiO_2_ molar ratio can affect the electrical properties inside the semiconducting grains, which will be discussed in the last section. Nevertheless, according to the impedance spectroscopy, the variation in the colossal dielectric properties and dielectric behavior of the NYCTO + xTiO_2_ ceramics should be described in all aspects based on the IBLC model.

**Figure 4:**
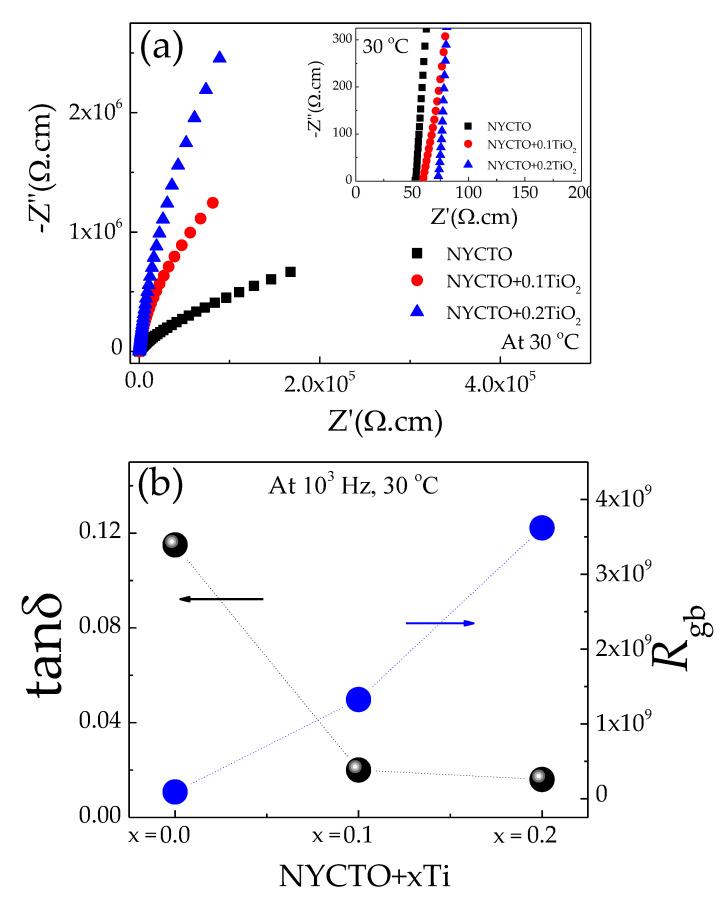
(**a**) Impedance complex plane (Z*) plots of NYCTO + xTiO_2_ ceramics at 30 °C; inset shows the enlarged view near the origin, showing a nonzero intercept. (**b**) Relationship of loss tangent (tanδ) at 1 kHz and 30 °C and grain boundary resistance (*R*_gb_) at 30 °C.

Figure 4a displays the Z* plots of all the ceramics at 30 °C. Only parts of the relatively large arcs can be observed with a nonzero intercept (inset of Figure 4a), which is similar to that reported in the previous works [5,6,12,19,21,44,54]. The *R*_g_ value at 30 °C, which can be calculated from the nonzero intercept, increases with increasing the excessive TiO_2_ molar ratio. Even though an entire arc cannot be observed, the *R*_gb_ values of all the NYCTO + xTiO_2_ ceramics can be estimated. As shown in Figure 4b, the tanδ of the NYCTO + xTiO_2_ ceramics is inversely proportional to the *R*_gb_ value. According to the IBLC structure [20,44], the low—frequency tanδ value is correlated to the total resistance, which is governed by the *R*_gb_ value. The low—frequency tanδ can be reduced by increasing *R*_gb_. Thus, the correlation of the tanδ and *R*_gb_ values follows the IBLC model.

**Figure 5:**
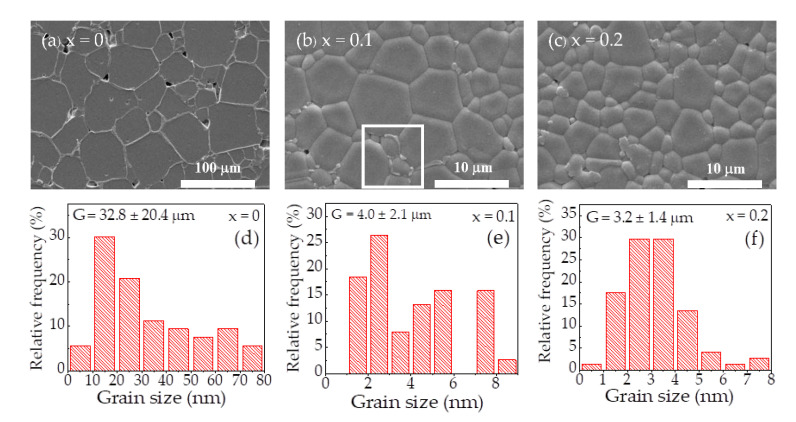
SEM images of NYCTO + xTiO_2_ ceramics with *x* = (**a**) 0.0, (**b**) 0.1 and (**c**) 0.2 and grain size distributions of NYCTO + xTiO_2_ ceramics with *x* = (**d**) 0.0, (**e**) 0.1 and (**f**) 0.2.

In addition to the variation in tanδ, the IBLC model should be used to reasonably describe the overall dielectric properties of the NYCTO + xTiO_2_ ceramics. Therefore, the microstructure of the sintered ceramics was studied. Figure 5 shows the SEM images of the polished surface of the NYCTO + xTiO_2_ ceramics and their grain size distributions. All the ceramics reveal the grain and GB structure with a highly dense microstructure. The mean grain size of the NYCTO + xTiO_2_ ceramics was extremely reduced by increasing x from 0 to 0.2. This result is similar to that reported in the previous reports for TiO_2_—rich CCTO ceramics [6]. The mean grain sizes of the NYCTO + xTiO_2_ ceramics with *x* = 0, 0.1 and 0.2 are 32.80 ± 20.44, 4.05 ± 2.08 and 3.18 ± 1.35 μm, respectively. The excessive TiO_2_ could intercept the grain growth rate of the NYCTO ceramics due to the pinning effect of excessive TiO_2_—rich phase particles during the sintering process [55]. We also found that the segregation of the Cu—rich phase is slightly observed along the grain boundaries, as remarked in the square area of Figure 5b.

**Figure 6:**
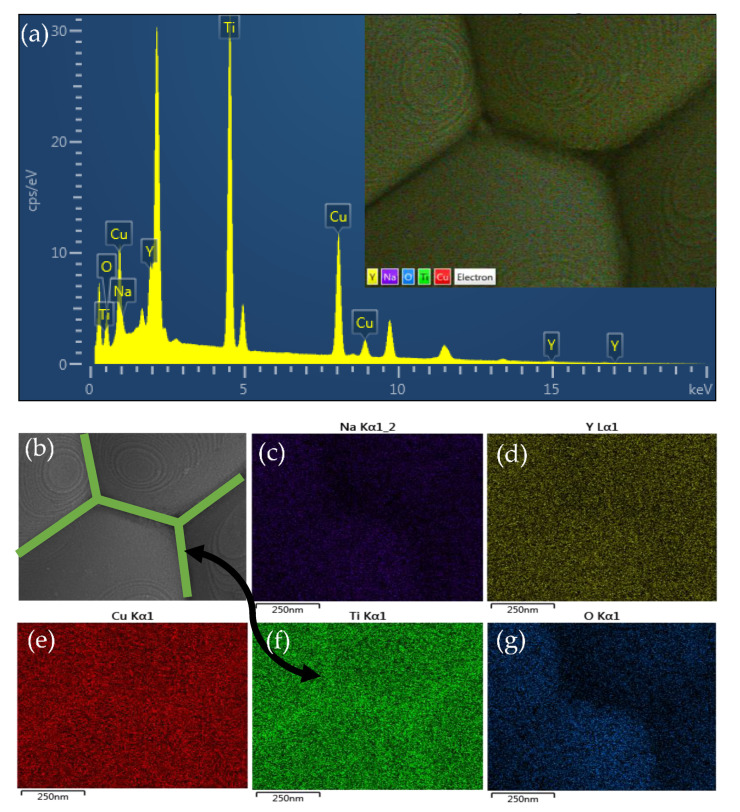
(**a**) EDS spectrum of NYCTO + xTiO_2_ ceramic with *x* = 0.1; inset shows EDS testing area. (**b**) SEM image and corresponding SEM—EDS mapping images of (**c**) Na, (**d**) Y, (**e**) Cu, (**f**) Ti and (**g**) O for NYCTO + xTiO_2_ ceramic with *x* = 0.1.

**Figure 7:**
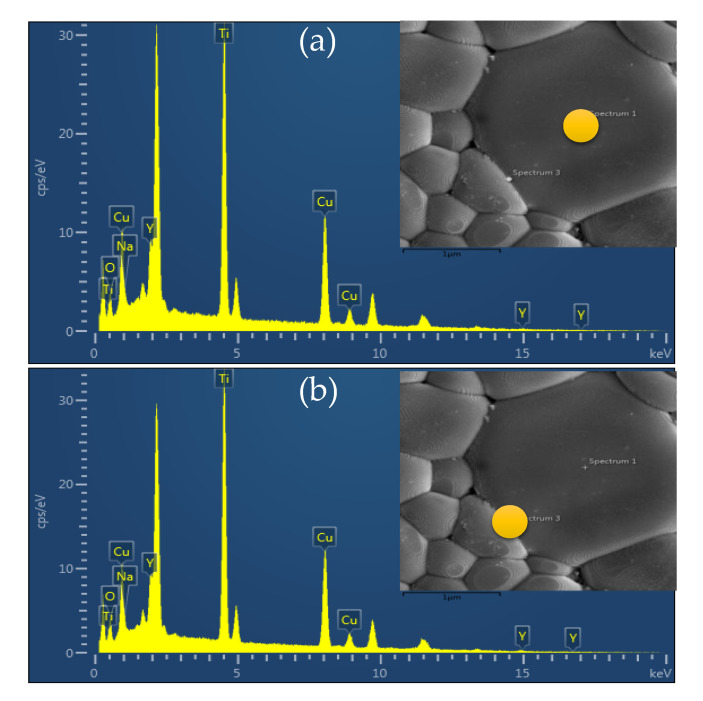
EDX spectra of NYCTO + xTiO_2_ ceramic with *x* = 0.2 detected at (**a**) grain and (**b**) GB; insets show the detected points in the microstructure.

The observed decrease in *ε*′ (Figure 2a) value of the NYCTO + xTiO_2_ ceramics should be caused by the decrease in the mean grain size, following a simple series layer model of the IBLC structure [20,44,56],

(1) $$\varepsilon^{\prime }=\frac{\varepsilon_{gb}^{\prime }G}{t_{gb}}$$

where *G* is the mean grain size, *t*_gb_ is the thickness of the GB and *ε*′_gb_ is the dielectric constant of the GBs. Furthermore, it is also suggested that, but does not clearly prove, the decrease in the *ε*′ might be due to the increase in *t*_gb_ due to the TiO_2_–rich phase. The EDS and EDS–SEM mapping techniques were used to further characterize the microstructure and elemental distribution. As revealed in Figure 6a and its inset, all elements comprising the NYCTO + 0.1TiO_2_ ceramic are observed in the EDS spectra, confirming the existence of Na, Y, Cu, Ti and O elements. As demonstrated in Figure 6b–g, the Na, Y, Cu and O elements disperse well throughout the microstructure. It is observed that the relatively higher brightness of Ti mapping element along the GBs compared to that of the grains can be observed, confirming the segregation of TiO_2_—rich boundary. Thus, this is one of the most important factors contributing to the decrease in the *ε*′ values of the NYCTO + 0.1TiO_2_ and NYCTO + 0.2TiO_2_ ceramics. According to the microstructure analyses, the density of the insulating GB layers in the NYCTO + xTiO_2_ ceramics was significantly increased by increasing TiO_2_—rich phase owing to the decreased mean grain size [9,20]. Obviously, The significantly increased *R*_gb_ (Figure 4) is also attributed to the enhancement of insulating GB density. Furthermore, the increased *R*_gb_ is also due to the increase in *t*_gb_. The XEX spectra detected at the grain and GB areas are shown in Figure 7. It was found that the percentage ratios of Ti(wt%)/Cu(wt%) at the grain and GB were found to be 0.808 and 0.834, respectively.

**Figure 8:**
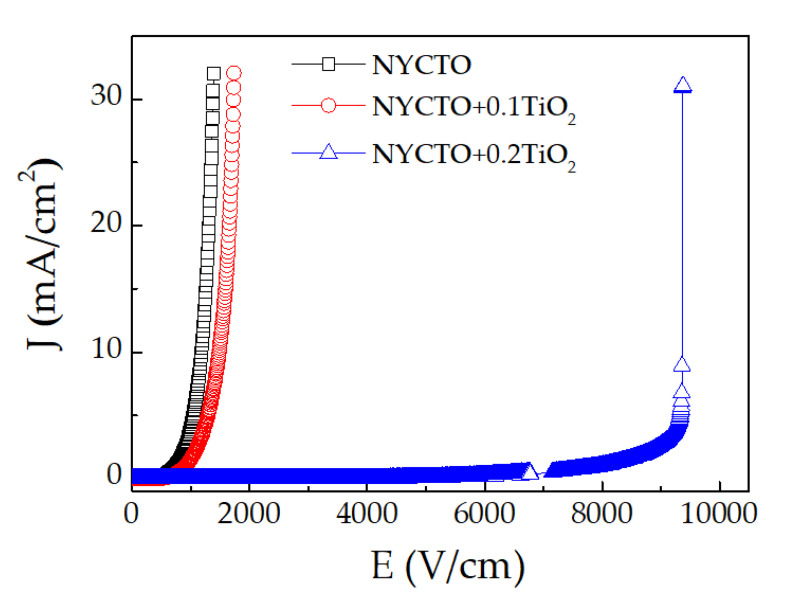
Nonlinear current density (*J*)—Electric field (*E*) at room temperature for NYCTO + xTiO_2_ ceramics with *x* = 0.0, 0.1 and 0.2.

According to our previous work [28,29], it was found that the NYCTO ceramics exhibited the nonlinear *J*–*E* characteristics with nonlinear coefficients (α) of 5.7–6.6. Furthermore, it was reported that the nonlinear properties of CCTO ceramics could be enhanced by increasing the excessive TiO_2_ molar ratio [6,7]. The α value of the CaCu_3_Ti_4+__x_O_12+2__x_ ceramic with *x* = 0.15 was increased to 7.9. As illustrated in Figure 8, all the NYCTO + xTiO_2_ ceramics exhibit the *J*–*E* characteristics. The α and electric breakdown (*E*_b_) of the NYCTO, NYCTO + 0.1TiO_2_ and NYCTO + 0.2TiO_2_ ceramics are 4.96 and 724.50 V/cm, 4.71 and 910.59 V/cm, and 13.36 and 7948.04 V/cm, respectively. The *E*_b_ increased significantly with increasing TiO_2_—rich phase, similar to that observed in the CaCu_3_Ti_4+__x_O_12+2__x_ ceramics [6]. Nevertheless, the enhanced α value of 13.36 is larger than that of the CaCu_3_Ti_4+__x_O_12+2__x_ ceramics. The segregation of TiO_2_—rich boundary and the increased GB density are the key factors, giving rise to the significantly improved nonlinear *J*–*E* properties. The α value is often related to *E*_b_ value [4].

**Figure 9:**
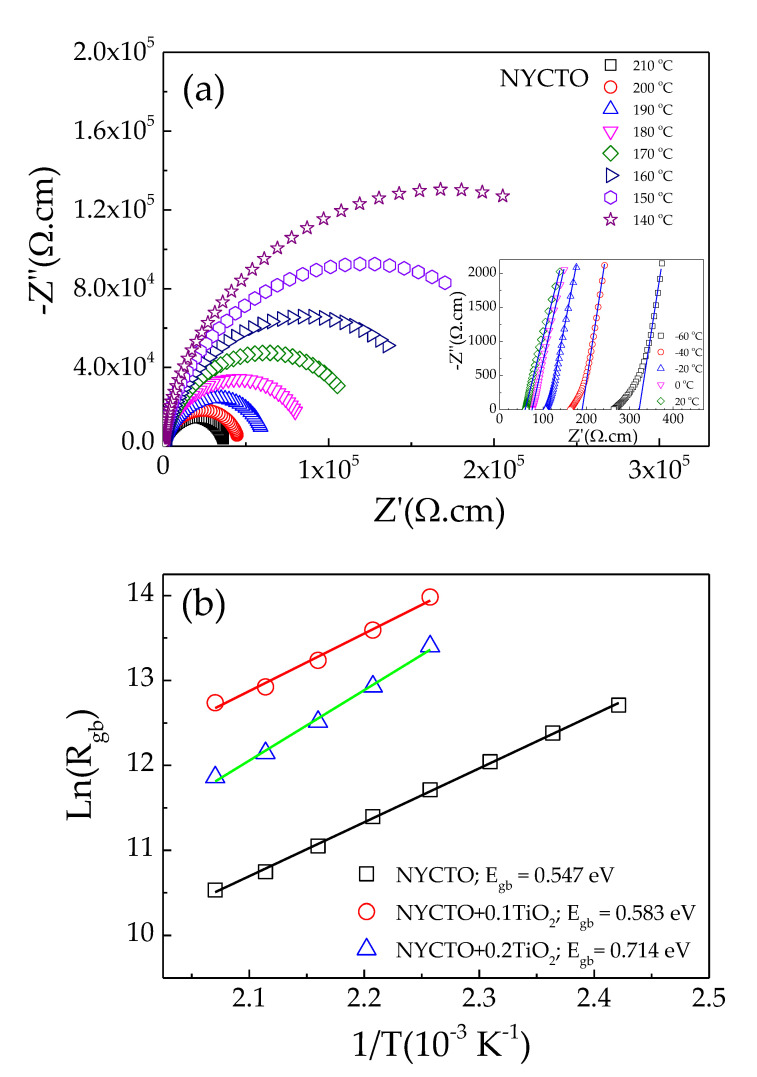
(**a**) Impedance complex plane plots (Z*) of NYCTO ceramic at various temperature (140–210 °C). (**b**) Arrhenius plot for grain boundary conductivity (*R*_gb_).

The nonlinear electrical behavior of ACu_3_Ti_4_O_12_ oxides is widely believed to be originated by the formation of the Schottky barrier at the GBs [4,5,7,18]. The increased *E*_b_ value is associated with the increase in *R*_gb_ due to the significantly increased GB density and GB thickness, which are classified as the geometric factors of the GBs [9]. Furthermore, the intrinsic factor of the GBs, i.e., the Schottky barrier height (Φ_b_), can usually have a remarkable effect on the *R*_gb_ and *E*_b_ values [4,5,7,9]. The Φ_b_ is closely related to the conduction activation energy at the GBs (*E*_gb_) [7,13,14]. To calculate the *E*_gb_ value, *R*_gb_ values at different temperatures were calculated. Figure 9a and its inset show Z* plots and nonzero intercept of the NYCTO ceramic at various temperatures. The *R*_g_ and *R*_gb_ values can be obtained and found to decrease with increasing temperature. Thus, E_gb_ can be calculated by using the Arrhenius law [5,9]:

(2) $$R_{gb}=R_{0}exp\left(\frac{E_{gb}}{k_{B}T}\right),$$

where *R*_0_, *k*_B_ and *T* are the per-exponential constant term, Boltzmann constant and absolute temperature, respectively. Figure 9b depicts the relationship between *R*_gb_ and 1000/*T* of the NYCTO + xTiO_2_ ceramics. The *E*_gb_ values can be calculated from the slopes of the *R*_gb_ and 1000/*T* plots, which are linearly fitted by using the Arrhenius law. The *E*_gb_ values of the NYCTO + xTiO_2_ ceramics are 0.547, 0.583 and 0.714 eV, respectively. Therefore, Φ_b_ of the NYCTO + xTiO_2_ ceramics can be increased by increasing the TiO_2_—rich phase. The improved nonlinear *J*–*E* properties and electrical properties of the GBs are also caused by the enhanced Φ_b_, just as observed in the TiO_2_—rich CCTO ceramics [7]. The increased Φ_b_ values of the NYCTO + xTiO_2_ ceramics are likely attributed to the suppressed oxygen vacancies and/or oxygen enrichment at the GBs due to the segregation of TiO_2_—rich boundary [7,18].

**Figure 10:**
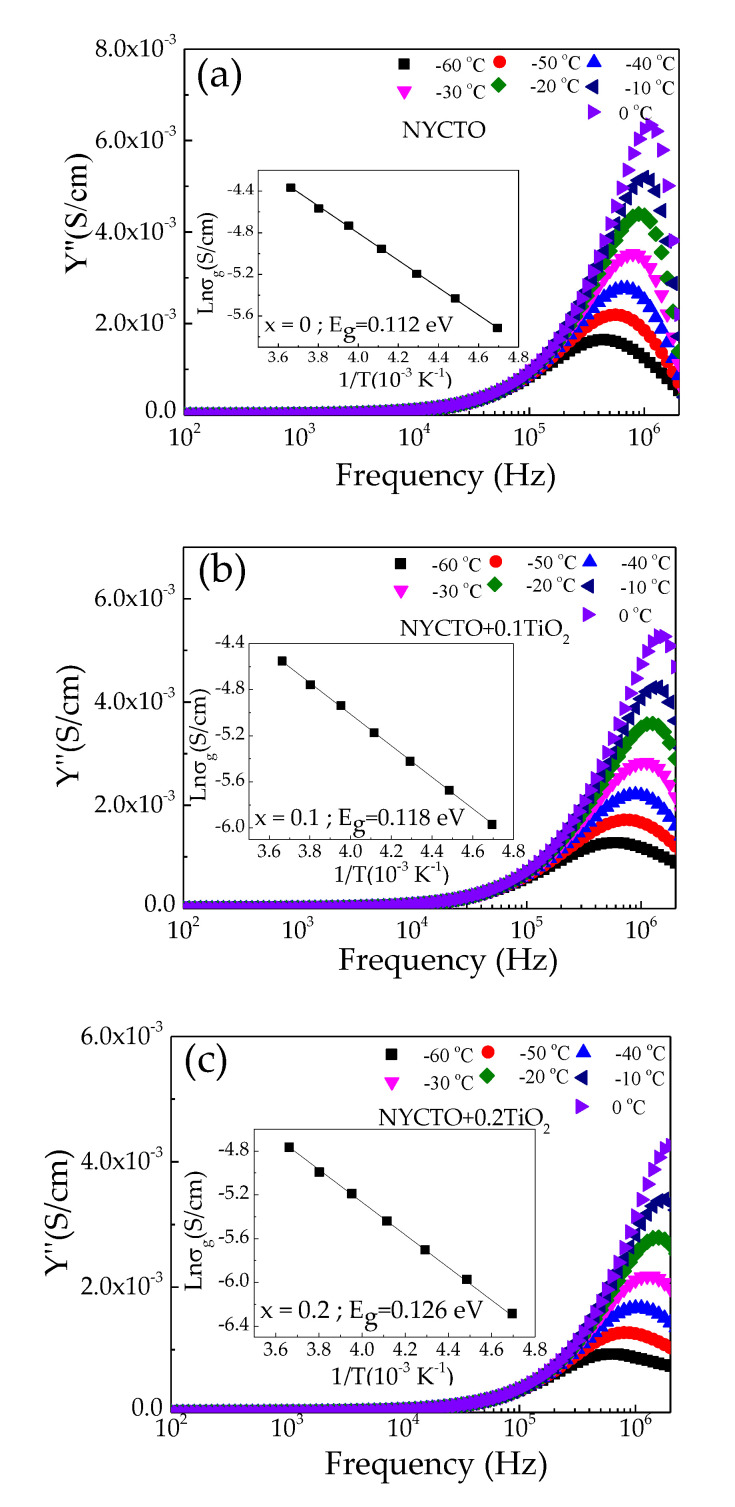
Imaginary part of admittance (Y″) as a function of frequency at different temperatures (−60–0 °C) for NYCTO + xTiO_2_ ceramics with *x* = (**a**) 0.0, (**b**) 0.1 and (**c**) 0.2; their insets show the Arrhenius plots for the grain conductivity (σ_g_).

In addition to the electrical properties of the GBs, the electrical properties of the semiconducting grains must also be characterized. The conduction activation energy of the grains (*E_g_*) can be calculated from the temperature dependence of *R*_g_. The *R*_g_ values at a low–temperature range can be easily calculated using the admittance spectroscopy (Y*) [28,29,57], as the following equation:

(3) $$Y^{*}=\frac{\left(R_{gb}^{-1}\right)\left(1-\omega^{2}\tau_{g}\tau_{gb}+i\omega \tau_{gb}\right)}{1+i\omega \tau },$$

where *τ_gb_* = *R*_gb_*C*_gb_, *τ_g_* = *R*_g_*C*_g_ and *τ* = *R*_g_*C*_gb_ and *C*_g_ and *C*_gb_ are the grain and GB capacitance values, respectively. According to the impedance spectroscopy, it was found that *R*_gb_ >> *R*_g_ and *C*_gb_ >> *C*_g_. From Equation (3), *R*_g_ can be obtained from the relation $R_{g}=1/2Y_{max}^{\prime \prime }$, where $Y_{max}^{\prime \prime }$ is the maximum value at $Y^{\prime \prime }$ peak. As shown in Figure 10a–c, $Y_{max}^{\prime \prime }$ appears in the temperature range from −60 to 0 °C. Consequently, *E_g_* can be calculated by using the Arrhenius law, $\sigma_{g}=\sigma_{0}exp\left(-E_{g}/k_{b}T\right)$, where σ_g_ is the grain conductivity ($\sigma_{g}$∝$1/R_{g}$), and σ_0_ is a per–exponential constant term. The *E*_g_ can be calculated by the linear fitting data, as demonstrated in the insets of Figure 10a–c. The *E*_g_ values are 0.112, 0.118 and 0.126 eV for the NYCTO, NYCTO + 0.1TiO_2_ and NYCTO + 0.2TiO_2_ ceramics, respectively. The *E*_g_ slightly increased with increasing the excessive TiO_2_, corresponding to the increase in *R*_g_ (Figure 3c). The difference between *E*_g_ and *E*_gb_ clearly indicates the formation of IBLC microstructure, consisting of the semiconducting grains and insulating GBs.

**Figure 11:**
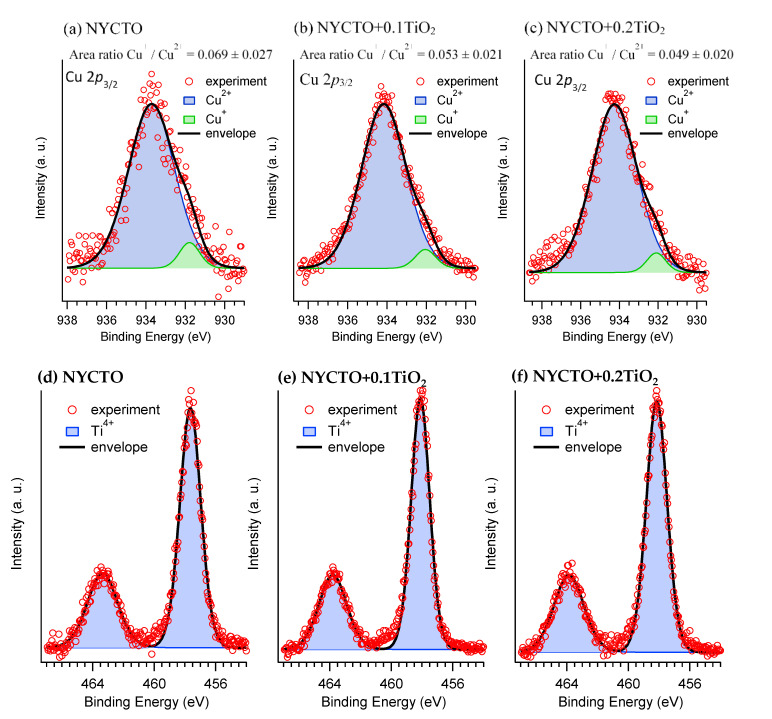
(**a**–**c**) XPS spectra of Cu 2*p*3/2 for all sintered NYCTO + xTiO_2_ ceramics. (**d**–**f**) XPS spectra of Ti 2*p* for all sintered NYCTO + xTiO_2_ ceramics.

The XPS technique was further used to characterize the electrical properties of the grains. Figure 11a–c displays the XPS spectra of the Cu 2*p*_3/2_ for the NYCTO, NYCTO + 0.1TiO_2_ and NYCTO + 0.2TiO_2_ ceramics, respectively. According to the fitted curves, the XPS peak of the Cu 2*p*_3/2_ can be divided into two peaks at relatively low and high binding energies, corresponding to the Cu^+^ and Cu^2+^, respectively. Note that only Ti^4+^ can be detected in the XPS spectra, as shown in Figure 11d–f. Thus, the conduction in the semiconducting grains of all the ceramics is attributed to the electron hopping between Cu^+^ ↔ Cu^2+^. The Cu^+^/Cu^2+^ ratios of the NYCTO, NYCTO + 0.1TiO_2_ and NYCTO + 0.2TiO_2_ ceramics are 0.069 ± 0.027, 0.053 ± 0.021 and 0.049 ± 0.020, respectively. The Cu^+^/Cu^2+^ ratios decreased with increasing TiO_2_—rich phase. Generally, CCTO and related ACu_3_Ti_4_O_12_ ceramics lose small amount of oxygen during sintering, giving rise to oxygen vacancies and associated free electrons [5,9]. Accordingly, a small amount of Cu^+^ and/or Ti^4+^ can be detected. For the NYCTO + 0.1TiO_2_ and NYCTO + 0.2TiO_2_ ceramics, the diffusion of oxygen vacancies during the sintering process may be inhibited by the segregation of TiO_2_—rich phase along the GBs. Thus, the oxygen loss and related oxygen vacancies in the TiO_2_—rich NYCTO ceramics were reduced, leading to the decrease in Cu^+^ ions. The increased *R*_g_ (Figure 3c) can be proved to be caused by the decreased Cu^+^/Cu^2+^ ratios. In addition to the grain size effect, the observed decrease in the *ε*′ of the NYCTO + 0.1TiO_2_ and NYCTO + 0.2TiO_2_ ceramics can be described based on the IBLC microstructure. Under al applied electric field, charge carriers inside the semiconducting grains are moved to trap at the insulating GB due to a high potential barrier height, inducing the interfacial polarization and hence high *ε*′ value. The intensity of the interfacial polarization of the NYCTO + 0.1TiO_2_ and NYCTO + 0.2TiO_2_ ceramics should be lower than that of the NYCTO ceramic because of a lower concentration of free carriers inside the grains, which is considered by a larger *R*_g_ value.

## 4. Conclusions

In conclusion, we have successfully synthesized the TiO_2_—rich NYCTO ceramics prepared using the SSR method. The effects of Ti—excess on the microstructure, colossal dielectric properties and nonlinear *J*–*E* characteristics were studied. Only the main phase of the NYCTO structure was detected in the XRD patterns, which might be due to the presence of an amorphous phase of TiO_2_ along the GBs. Significantly reduced grain with highly dense microstructure was observed in the TiO_2_—rich NYCTO ceramics, which was due to the pinning effect of the TiO_2_—rich phase particles. The reduced grain sizes, which can cause an increase in the GB density, resulted in the significant enhancement of R_gb_, and hence reduced tanδ. The colossal *ε*′ values of ~ 0.7–1.4 × 10^4^ was achieved in the TiO_2_—rich NYCTO ceramics. The TiO_2_—rich NYCTO ceramics also showed the enhanced nonlinear *J*–*E* properties due to the improved GB properties. The R_g_ value was also increased owing to the decreased Cu^+^/Cu^2+^ ratio, confirming by the XPS result. The overall colossal dielectric permittivity and nonlinear electrical properties were well described using the Maxwell–Wagner polarization relaxation model based on the formation of the Schottky barrier at the grain boundary.

## Data Availability

The data presented in this study are available in article.
